# An Update on Dyslipidemia Management and Medications: A Review

**DOI:** 10.7759/cureus.56255

**Published:** 2024-03-16

**Authors:** Ziad A Taher, Abdulrahman A Taher, Suhaib Radi

**Affiliations:** 1 Department of Medicine, King Abdulaziz Medical City, Jeddah, SAU; 2 College of Medicine, University of Jeddah, Jeddah, SAU; 3 College of Medicine, King Saud Bin Abdulaziz University for Health Sciences, Jeddah, SAU; 4 College of Medicine, King Abdullah International Medical Research Center, Jeddah, SAU

**Keywords:** pcsk9 inhibitors, inclisiran, cardiovascular diseases, hypercholesterolemia, dyslipidemia

## Abstract

Dyslipidemia, characterized by abnormal lipid levels in the bloodstream, is a very common and underappreciated chronic disease associated with a significant cardiovascular disease burden. The management landscape for dyslipidemia has historically been static, with a sparse selection of therapeutic options. This article presents a comprehensive review of contemporary approaches to dyslipidemia management, focusing on therapeutic strategies and emerging interventions. We delineate the most current American Heart Association/American College of Cardiology & Canadian Cardiovascular Society guidelines and examine pivotal clinical trials that are shaping the contemporary approach to dyslipidemia management.

## Introduction and background

Dyslipidemia and its management were a relatively stagnant topic with few updates; however, this is no longer the case. It is currently one of the medical areas that is changing the fastest because of new drugs and updated recommendations from societies and communities around the world. The Food and Drug Administration has authorized more medications in the last 10 years, and standards are being modified more frequently than in the past [1].

In order to give current updates for clinical practice, we have reviewed the most commonly accepted papers and the most recently published research regarding dyslipidemia.

## Review

Types of lipids and clinical significance

Any sort of lipid abnormality in the blood can be referred to as dyslipidemia. As a result, the most common conditions affecting the following lipid types will be covered in this article: (i) Low-density lipoprotein-cholesterol (LDL-C); (ii) high-density lipoprotein-cholesterol (HDL-C); (iii) cholesterol; (iv) triglycerides.

Lipids are insoluble in plasma, for that, it is transported by proteins called lipoproteins which are divided into four main classes as presented in Table 1.

**Table 1:** Lipoprotein Classes and Functions TAG: Triglyceride; VLDL: very low-density lipoprotein; IDL: intermediate lipoprotein; LDL: low-density lipoprotein; HDL: high-density lipoprotein

Function	Source	Class
Transport dietary TAG	intestine	Chylomicrons
Transport of endogenously synthesized TAG	liver	VLDL
Transport of cholesterol to peripheral tissue	Blood circulation	IDL & LDL
Transport cholesterol from peripheral tissue to liver	liver	HDL

One of the main risk factors for heart disease is dyslipidemia. LDL-C and triglycerides are the two primary dyslipidemia-related factors that directly affect cardiovascular health, according to the American Heart Association. The risk of atherosclerotic cardiovascular disease (ASCVD) is higher in those with elevated LDL-C levels. Elevated levels of triglycerides are linked to a higher chance of developing cardiovascular disease and pancreatitis [1].

LDL-C remains the targeted atherogenic lipoprotein in most guidelines. However, over the last few years, the importance of other lipoproteins has come into play including non-HDL-C, ApoB, and lipoprotein (a) [Lp(a)] as accurate indicators of CV mortality. These parameters offer a superior assessment of atherogenic particles compared to LDL-C, particularly noteworthy in patients with TG > 1.5 mmol/L [2-4].

Familial hypercholesterolemia (FH)

FH is a common genetic disorder affecting children and adults, marked by elevated levels of LDL-C that contribute to ASCVD. Early detection is crucial for effective treatment. Mutations in three genes, LDL receptor (LDL-R), proprotein convertase subtilisin/kexin type 9 (PCSK9), and apo B, can cause FH. However, most FH cases involve LDL-R mutations, reducing lipid uptake in the liver and increasing circulation, a primary cause of ASCVD and mortality. There are two main types of FH: homozygous FH, rare and associated with LDL-C level >13 mmol/l, posing a risk of death before age 20 if untreated; and heterozygous FH, characterized by >4.9 mmol/l LDL-C level, with ASCVD development likely before the age of 60. Family cascade screening is recommended for all family members of the affected person including children when they reach the age of 10 years or maybe earlier at the age of 2 years [5,6].

In the treatment of FH, the objective is to reduce LDL-C levels to below 2.6 mmol/L or achieve a minimum reduction of 50%. Typically, statins serve as the primary medication, often followed by the addition of ezetimibe, colevabsam (a bile acid sequestrant), PCSK9 inhibitors, and lomitapide (microsomal triglyceride transfer protein). When these treatments prove insufficient, LDL apheresis may be considered as a last resort [7].

When to treat?

The primary approach for managing dyslipidemia has remained the consistent use of statin therapy. Additional medications may be considered if the target levels are not achieved or if the patient cannot tolerate statin therapy. It is crucial to highlight the following indications for therapy: (i) LDL-C ≥5 mmol/L, OR ApoB ≥1.45 g/L, OR non-HDL-C ≥5.8 mmol/L OR confirmed familial hypercholesterolemia; (ii) Diabetic patients older than 40 years OR those older than 30 years with a DM history of more than 15 years OR those with microvascular complications; (iii) Chronic kidney disease with an estimated glomerular filtration rate <60 mL/min/1.73 m2, persistent for three or more months, OR an albumin-to-creatinine ratio >3 mg/mmol AND ≥50 years; (iv) As primary prevention for patients with a high atherosclerotic cardiovascular risk score (initiating treatment when necessary); (v) As secondary prevention in patients with any atherosclerotic condition, including cardiac disease, cerebrovascular disease, peripheral artery disease, and aneurysms; (vi) A recent meta-analysis recommended maintaining lower lipid levels for elderly adults >75 who don't meet the above indications, as it has shown to yield beneficial outcomes compared to others [8].

Treatment and management of dyslipidemia

The primary approach to treating dyslipidemia involves lowering LDL-C and increasing HDL-C levels. This is typically achieved through lifestyle modifications, including diet, weight loss, and exercise. However, these interventions may vary among individuals based on several factors. If LDL-C levels remain unchanged after six months, drug therapy should be considered as part of the treatment plan.

Diet Therapy

The objective of diet therapy is to minimize cholesterol intake and typically involves three stages. Initially, the goal is to reduce daily cholesterol intake to below 300mg/day. Subsequently, the intake is further reduced to less than 200mg/day. Finally, cholesterol intake is restricted to 25 mg/day. Additionally, exercise plays a crucial role not only in reducing body weight but also in increasing HDL-C levels. Individuals with hypercholesterolemia are advised to engage in 30 to 60 minutes of moderate to vigorous aerobic exercise, at least three days per week, which significantly contributes to preventing coronary artery disease [9].

Drug Therapy

Drug therapy is an essential adjunct to dietary modifications to lower LDL-C levels. While diet therapy typically takes 3 to 6 months to show results, drug therapy tends to have a quicker impact. If one drug fails to lower LDL-C, considering add-on drug therapy is advisable. However, it's important to note that many lipid-lowering agents can have various side effects. Here's a discussion of the various classes of drugs approved for the management of dyslipidemia:

HMG-CoA Reductase Inhibitors (Statins)

Statins, also known as HMG-CoA reductase inhibitors, are widely tolerated and commonly prescribed medications. They function by inhibiting HMG-CoA reductase, a rate-limiting enzyme in cholesterol synthesis, leading to a decrease in cholesterol levels within hepatocytes. As a compensatory response, cell wall surface receptors increase to meet the cellular need for cholesterol, resulting in decreased blood LDL-C levels [10]. Furthermore, a meta-analysis study revealed that in addition to reducing serum LDL-C levels, it was linked to the regression of plaque in patients with coronary atherosclerosis, as assessed by intracoronary ultrasound [11].

Statins can also reduce triglycerides by 10-20% and minimally increase HDL by around 5-10% [12]. Side effects of statin therapy have been extensively studied, with the most common being myalgia occurring in approximately 1-10% of cases. Rhabdomyolysis, a more serious side effect, is rare, occurring in less than 0.1% of cases [13]. Other reported side effects include hepatic dysfunction and renal impairment secondary to rhabdomyolysis. Groups more prone to these side effects include the elderly, females, hypothyroid patients, and those with multisystem diseases.

For patients unable to tolerate these side effects, switching to ezetimibe is recommended. In cases where there is a high risk of cardiovascular complications, consideration might be given to shifting to another type of statin therapy with dose adjustments or less frequent dosing, such as twice weekly. Statins remain a Class I recommendation in most of the major international guidelines including the American Heart Association and the European Society of Cardiology for patients with acute coronary syndrome [14,15].

Ezetimibe

Ezetimibe can enhance the efficacy of statin therapy for individuals at high risk of cardiovascular events, decreasing the incidence of non-fatal heart attacks and strokes [16,17]. The 2021 CCS dyslipidemia guidelines underscore its significance in reducing cardiovascular mortality, recommending its use as a secondary treatment alongside statin therapy for lowering cardiovascular risk in both primary and secondary prevention [18].

Ezetimibe operates by hindering cholesterol absorption in the intestine, likely through the inhibition of the NPC1L1 protein. This results in a decrease in the serum levels of lipoprotein cholesterol transported to the liver. As a compensatory mechanism, there is an increase in LDLR receptors in the liver, leading to enhanced clearance of cholesterol from the circulation. This process results in a 20% reduction in LDL-C levels.

The efficacy of ezetimibe was demonstrated in the IMPROVE IT trial, conducted in 2015 with 18,144 participants. The trial aimed to assess the reduction in cardiovascular death, non-fatal myocardial infarction, and unstable angina. Notably, Ezetimibe exhibited improved cardiovascular outcomes when initiated in acute coronary syndrome patients with LDL-C levels exceeding 1.3 mmol/l [17].

Bile-Acid Sequestrants

The mechanism of bile-acid sequestrants is contingent on bile-acid levels in the liver. By reducing bile acids after binding to resins in the small intestine and being excreted in stool, LDL receptor numbers increase. This stimulates the production of more bile acid from circulating cholesterol, ultimately decreasing LDL-C levels in the blood [17]. Common side effects include constipation, reflux esophagitis, and nausea. Additionally, it can reduce the absorption of various drugs like digitalis, beta-blockers, and warfarin. Bile-acid sequestrants typically lower LDL-C by 15-30% and increase HDL-C by 3-5%.

Niacin

Niacin, or nicotinic acid, is a water-soluble vitamin with notable effects on LDL-C, triglycerides, and HDL-C. While it is the most effective in raising HDL-C among dyslipidemia drugs, it's not as potent as statins in lowering LDL-C. Niacin decreases the mobilization of fatty acids from adipose tissue, leading to a drop in triglycerides and very low-density lipoprotein cholesterol synthesis [19]. However, its use is limited due to common side effects like flushing, pruritus, rash, nausea, and dyspepsia, causing discontinuation in 10%-50% of patients. An extended-release form with better tolerance is Food and Drug Authority (FDA)-approved. Despite its withdrawal for cardiovascular disease prevention, niacin is still used for other FDA-approved indications [3,4].

Fibrates

Fibrates, another lipid-lowering agent, primarily impact triglycerides. They do not show any cardiovascular mortality benefit but can decrease the complications related to very high levels of triglyceride like pancreatitis [20]. Fibrates can reduce triglycerides by 50% compared to omega-3 fatty acids. To a lesser extent, they lower LDL-C levels and increase HDL-C levels. Fibrates activate peroxisome proliferator-activated receptors, transcribing genes involved in peroxisomal B-oxidation. Common side effects include elevated liver enzymes and creatine phosphokinase, along with myopathy, cholelithiasis, and venous thrombosis to a lesser extent. Certain fibrates (gemfibrozil) should not be used concurrently with statin therapy, as they can inhibit statin metabolism, increasing the risk of myopathy [21,22].

Omega-3 Fatty Acids (FAs)

Similar to fibrates, omega-3 FAs primarily impact triglycerides, reducing levels by 25%-30%. They are approved by the American Heart Association for use in hypertriglyceridemia. However, certain forms of omega-3 FAs may increase LDL-C levels. Despite extensive clinical trials, Omega-3 FAs have failed to demonstrate a mortality benefit in cardiovascular patients. Trials like ASCEND and STRENGTH, involving large sample sizes, did not show cardiovascular benefits [23,24]. The STRENGTH trial was terminated early due to the absence of cardiovascular benefits and common gastrointestinal side effects in the active treatment group. Unfortunately, the exact mechanism of action for Omega-3 FAs in triglyceride reduction is not well understood [25].

It's intriguing that despite the association between hypertriglyceridemia and increased ASCVD events, omega-3 FAs and other triglyceride-lowering agents like fenofibrate have not demonstrated a decrease in ASCVD morbidity or mortality. As a result, their approval is limited to cases of severe hypertriglyceridemia (>500-1000) to prevent pancreatitis.

The controversy surrounding this may be attributed to the possibility that omega-3 FAs were used in less effective doses or the use of different forms, such as eicosapentaenoic acid (EPA) and/or docosahexaenoic acid. This highlights the importance of understanding the nuances in dosages and specific formulations when studying the effectiveness of these agents in preventing cardiovascular events [26].

The REDUCE-IT trial focused on studying icosapent ethyl, a form of omega-3 FA, administered at a dosage of 2 g twice daily with meals. This trial spanned five years and included nearly 5000 participants. The findings revealed that individuals in the active treatment group were 25% less likely than the comparison group to develop unstable angina, undergo coronary revascularization, experience non-fatal myocardial infarction, and suffer a stroke [27].

Following the positive outcomes from the REDUCE-IT trial, the FDA granted approval to EPA in December 2019 for use in patients with triglyceride levels ≥150 mg/dL, established cardiovascular disease or diabetes, and two or more additional risk factors for cardiovascular disease. This underscores the potential of specific formulations and dosages of omega-3 FAs in reducing cardiovascular events in high-risk individuals [28].

PCSK9 Inhibitors

The development of PCSK9 inhibitors indeed represents a revolutionary advancement in dyslipidemia treatment. These synthesized antibodies target proprotein convertase subtilisin/kexin type 9 (PCSK9), an enzyme responsible for breaking down LDL-C receptors in hepatocytes. Typically, PCSK9 works to enhance the removal of LDL-R from the blood, leading to increased LDL-C levels [29].

Inhibiting this enzyme has been shown to reduce LDL-C levels by 45% to 60%, regardless of whether statin therapy is also utilized. Notable agents in the PCSK9 inhibitor class include alirocumab and evolocumab. The FOURIER trial, involving 27,564 participants and assessing the cardiovascular benefits of evolocumab alongside maximally tolerated statin therapy in patients with ASCVD, concluded that while there was a reduction in composite cardiovascular outcomes, there was no significant impact on cardiovascular and all-cause death. This underscores the complexity of evaluating the overall mortality benefits of these innovative treatments [30].

The ODYSSEY OUTCOME trial, conducted similarly for alirocumab, reached the same conclusion as the FOURIER trial, demonstrating a decrease in cardiovascular morbidity but not in mortality. Both trials provided evidence of the positive impact of PCSK9 inhibitors on composite cardiovascular outcomes [31].

As for side effects, injection-site reactions are noted as the primary adverse effect of PCSK9 inhibitors. Remarkably, these drugs, being fully human monoclonal antibodies, rarely trigger immunologic responses such as allergies or the formation of antidrug-neutralizing antibodies. This aspect contributes to the safety profile of PCSK9 inhibitors in their role as innovative lipid-lowering agents [29].

Inclisiran

Inclisiran, a novel agent approved by the FDA in July 2023, is indicated for use in patients with high ASCVD risk and elevated LDL levels while on statin therapy, as well as for patients with heterozygous FH. It operates as a small interfering RNA that inhibits the production of the PCSK9 enzyme in hepatocytes. As previously mentioned, PCSK9 plays a critical role in the destruction of LDL-C receptors in hepatocytes, which are responsible for the uptake of LDL-C from the blood [32].

Notably, inclisiran was initially available in a less stable form, requiring administration through an intravenous line. However, advancements have led to the development of a more stable form, allowing for subcutaneous administration every three months, followed by subsequent doses every six months. This marks a significant improvement in the convenience and accessibility of the treatment [33].

Insights from the ORION-10 and ORION-11 trials revealed an approximately 50% reduction in LDL-C levels in patients with ASCVD or at high risk of ASCVD while on the maximal statin therapy dose after 90 days [34,35].

While these results are promising in terms of LDL-C reduction, it is important to note that the studies have not confirmed any cardiovascular morbidity or mortality benefits yet. Ongoing research and further studies will likely provide a more comprehensive understanding of Inclisiran's impact on cardiovascular outcomes [36].

Bempedoic Acid

Bempedoic acid, a recently developed therapeutic agent, has emerged as a promising addition to the armamentarium for managing hypercholesterolemia. Unlike traditional statin therapy, which targets HMG-CoA reductase, bempedoic acid acts upstream in the cholesterol biosynthesis pathway by inhibiting ATP citrate lyase. By disrupting cholesterol production at this early stage, bempedoic acid effectively reduces circulating levels of LDL-C, thereby mitigating the risk of atherosclerotic cardiovascular disease.

CLEAR wisdom and CLEAR harmony trials, which assessed the efficacy and safety of bempedoic acid, have demonstrated its efficacy in LDL-C reduction as monotherapy and in combination with other lipid-lowering agents. It showed reduction in the LDL-C level by 13.9 to 17.4 % when used with a dose of 180 mg alone. When combined with 10 mg of ezetimibe the result was further dropped to 39%. Furthermore, bempedoic acid exhibits a favorable safety profile, with adverse effects comparable to placebo, making it a valuable option for patients intolerant to or inadequately controlled on statins and who cannot afford PCSK9 inhibitors. It was approved by the FDA in 2020 for use in hypercholesterolemia patients. Future research aims to explore its long-term cardiovascular benefits and its role in combination therapy with existing treatments, providing clinicians with additional tools for managing dyslipidemia and improving patient outcomes [37-39].

## Conclusions

Dyslipidemia is a very common and underappreciated metabolic abnormality globally, and more specifically, locally. There have been new and rapid advancements recently with the introduction of novel drugs showing improvement in patients’ outcomes. There are still some medications under clinical trials that might also change the practice in dyslipidemia over the next few years.
